# Supplementary material for: Are carbon emissions trading and green financial instruments synergistic? -Comprehensive quantitative research based on content analysis
Source: PLoS One. 2024 Mar 7;19(3):e0298601. doi: 10.1371/journal.pone.0298601 (PMC10919601; doi:10.1371/journal.pone.0298601)
Supplement: S1 Data — (ZIP) [file pone.0298601.s001.zip › Data/地级市Z_F工作报告.zip!/╔┬╬≈/║║╓╨╩╨/└┤╫╘╣1⁄2╓┌║┼í╛┬φ┐╦╩2╛▌═°í┐_2014.docx]

各位代表：

现在，我代表市人民政府向大会作工作报告，请予审议，并请市政协委员和其他列席人员提出意见。

一、2013年工作回顾

过去一年，在省委、省政府和市委的坚强领导下，全市上下以党的十八大精神为指针，围绕主题主线，坚持稳中求进，迎难而上，苦干实干，经济社会发展呈现出稳中有进、稳中向好、稳中提质的良好态势，较好地完成了四届人大四次会议确定的各项目标任务。

——主要经济指标较快增长。全年实现生产总值881.73亿元，增长12.7%。财政总收入83.75亿元、增长10.3%，地方财政收入35.83亿元、增长19.1%；财政支出214.4亿元、增长9.5%。城镇居民人均可支配收入22167元、增长11.8%，农民人均纯收入7053元、增长14.1%。

——三次产业协调发展。规模以上工业总产值856.1亿元、增长25.6%，增加值270.67亿元、增长22.2%。农业总产值305.07亿元、增长5.2%。社会消费品零售总额248.05亿元、增长14.9%。金融机构各项存款、贷款余额分别达到1246.83亿元、491.85亿元，增长16.2%、22.2%。三次产业结构为20.2∶45.1∶34.7，非公经济占生产总值比重为52.52%。

——项目建设成效显著。134个重点项目完成投资293亿元，占年计划的116.7%。全社会固定资产投资完成679.3亿元、增长27%。招商引资到位资金350亿元、增长10.6%。

——生态建设加快推进。全年造林23.9万亩，治理水土流失850平方公里。万元生产总值能耗下降3.5%，化学需氧量、氨氮、二氧化硫、氮氧化物削减完成省上下达指标。中心城区空气质量二级以上优良天数达350天，汉江出境水质始终保持二类标准。

——公共服务显著改善。全年民生类支出173.2亿元、占财政总支出的80.8%，社会保障和教育、卫生、科技、文化、体育等社会事业全面发展。陕南移民搬迁2.2万户8.36万人；保障性住房基本建成27055套；汉江综合整治建成堤防主体46.9公里。人民群众对社会治安的满意率位居全省前列。

一年来，我们着力抓了以下几方面工作：

（一）狠抓项目建设，发挥投资拉动作用。着眼政策导向、资源优势、长远发展谋划项目，坚持领导包抓、现场观摩、考核激励推进项目，通过高效服务、亲商安商、优化环境保障项目，汉中机场、十天高速略阳段、西汉高速佛坪连接线、汉中茶城等项目基本建成，陕钢汉中公司棒材生产线、中心医院门诊住院楼等项目建成投运，西成客专、宝汉高速、汉王药业等项目超额完成年度投资计划，洋县有色冶金产业园、108国道汉勉段改建等项目开工建设。

（二）强化产业支撑，多措并举提速增效。农业上，坚持扩张规模强产业、集成要素建园区、全力以赴促增收、创新体制增活力，实现了农村经济稳步增长。粮、油产量分别达102.7万吨、18.5万吨；生猪出栏404.8万头，茶叶、蔬菜、水果产量分别达2.6万吨、202.4万吨、41.2万吨，“汉中仙毫”荣获第31届巴拿马国际博览会金奖；省、市级园区新增66个，市级以上龙头企业达到154家。工业上，坚持狠抓稳产扩销、扶强扶优、技术改造、园区建设，保持了工业经济较快增长。新培育规模以上企业49户，陕钢汉中公司成为我市首家产值过百亿元企业；80个重点技改项目完成投资55亿元；工业园区建设完成投资22.8亿元，实现产值435亿元、增长25%，新增省级工业园区6个、陕西名牌产品16个，勉县成功创建“全国食品工业强县”。三产上，坚持整合资源、捆绑投入、重点倾斜、打造精品，推动了旅游商贸的有力增长。“一江两岸”景区建设有新突破，张骞墓申遗通过国际专家验收评估，龙岗遗址考古公园获国家审批立项，华阳景区“五百罗汉堂”、紫柏山景区滑雪场等项目建成投运，全年接待游客2249.8万人次、旅游收入106.1亿元，分别增长18%、29.1%。加快城乡市场体系建设，带动现代服务业快速发展。世纪阳光二期工程建成投用，褒河物流园新增入园企业3户；新培育限额以上企业62户，乡镇标准化超市、“万村千乡”农家店、农贸市场和“新网工程”建设取得新成效。

（三）加快城乡建设，全面提升城镇化水平。按照“做美城市、做强县城、做大集镇、做好社区”要求，统筹做好规划建设工作。开展了市域城镇体系、历史文化名城保护规划编制工作，县城、建制镇、新型农村社区、移民搬迁安置点规划进一步完善。中心城区30个重点基础设施项目完成投资18.7亿元，滨江新区、兴元新区、江南片区建设和老城区改造步伐加快，西新街西段、东排洪渠改造等工程建成投用，西二环、虎头桥路西段等项目加快推进。县城基础设施建设完成投资52.8亿元，省、市级重点镇完成投资21亿元，农村清洁工程完成年度投资4.53亿元。全市城镇化率43.12%，比上年提高1.62个百分点。深入开展建筑垃圾、市容环境等专项整治，绿化、净化、亮化、美化和交通管理水平进一步提升。汉中卫生城市创建通过国家爱卫办技术评估。

（四）突出循环发展，加快推进生态文明。全面开展节水节地节材和资源综合利用，淘汰了7条落后产能生产线，工业、交通、建筑、公共机构等重点领域实现节能目标。围绕总量削减和环境质量改善双达标，突出抓了42个污染减排治理项目。以秸秆禁烧为突破口，多管齐下、综合施策，大气污染防控取得显著成效。以开展植树造林年和城市绿化年活动为抓手，全面开展城乡绿化，宜林区域绿化率进一步提升。

（五）坚持以人为本，多方努力改善民生。全面落实支持就业的各项政策措施，新增就业4.6万人。大力推进教育优先发展，80所公办幼儿园基本建成，352所学校达到标准化要求，“营养计划”惠及25.8万学生，宁强、佛坪县“双高双普”通过省政府验收命名，全市高考二本以上上线人数增幅和上线率增幅全省第一。深入推进医药卫生和食品药品监管体制改革，县以下公立医疗机构全面实现药品零差率销售，新农合参合率99.8%，人均基本公共卫生服务经费提高到30元。稳定低生育水平，人口自然增长率2.69‰。大力开展科技创新，申请专利1403件，取得科技成果226项。不断完善城乡社保体系，城镇基本医保覆盖率、城乡居民养老参保率分别达99.6%、100%；新建区域性敬老院和县级养老服务中心7个、农村互助幸福院100个；城乡低保27.2万人，医疗救助、临时救助11.2万人，五保供养2.03万人。加大扶贫开发工作力度，全年脱贫13.76万人。深入实施文体惠民工程，大型舞台剧《汉颂》正式公演，村文化室、农家书屋等实现行政村全覆盖，完成了13.2万户广播电视“户户通”建设任务，文化产业增加值增长29.7%；全民健身活动广泛开展，成功举办市第五届运动会等系列赛事。全面履行稳定第一责任，安全生产事故起数、死亡人数分别下降28.2%、35%，“毒豆芽”、“毒馒头”等专项整治成效明显，信访“一推进、两下降、创三无”目标顺利实现，治安防控体系进一步完善，社会大局和谐稳定。

（六）严格正风肃纪，大力提升政府效能。认真贯彻中央八项规定，严格落实中省市关于停建楼堂馆所、清理办公用房、严禁公款送礼、厉行勤俭节约、规范公务接待和公车使用等一系列规定，全市“三公”经费支出较上年下降5.5%。政府工作人员深入一线摸实情、听民意、解民忧，持续改文风、转会风、抓落实，文件和会议数量分别下降28%和18%。自觉接受人大依法监督和政协民主监督，全年邀请省市人大代表、政协委员、专家教授211人次列席政府重要会议，及时听取对政府重要决策的意见建议；高度重视人大建议、政协提案办理，办结率100%，满意率98%以上；扎实推进政府信息和政务公开，深入开展公民代表进机关活动，市长信箱、市长热线、人民网地方领导留言办结率100%。充分发挥市县行政服务中心作用，全年审批事项办结率98.3%。

审计、工商、统计、供销、人防、物价、民族、宗教、档案、地震、气象、外事、侨务、对台、慈善、老龄、地方志、预备役和残疾人等各项工作都取得新成绩。

各位代表，过去一年是不平凡的一年，是全市上下抢抓机遇、应对挑战、实现经济逆势上扬的一年，是干部群众同心同德、凝心聚力、开创“三市”建设新局面的一年。成绩来之不易，这是省委、省政府和市委坚强领导的结果，是市人大、市政协和社会各界监督支持的结果，是全市广大干部群众改革创新、苦干实干的结果。在此，我代表市人民政府，向为汉中发展做出贡献的工人、农民、知识分子、干部、驻汉部队指战员、武警官兵和公安干警致以崇高的敬意！向大力支持政府工作的各位代表、各位委员和社会各界人士表示衷心的感谢！

回顾一年工作，我们也清醒地看到存在的问题，主要是：经济总量偏小，产业结构不合理，增长方式粗放，发展面临着增长与结构调整、方式转变的双重压力；财政收支矛盾突出，政府债务较多，市场融资难度加大，用地、资金等要素制约日益凸显，保持较快发展压力较大；城镇化率、经济外向度、居民收入水平低的问题依然存在，已成为发展中需要解决的突出问题；南水北调中线工程即将投运，生态主体功能区规划逐步实施，生态环境对发展的刚性约束进一步加大；少数干部责任心不强，担当意识不足，执行力有待进一步提升。这些问题，我们必须高度重视，认真加以解决。

二、2014年工作思路和目标任务

今年是贯彻党的十八届三中全会精神、全面深化改革的第一年，也是确保“十二五”目标圆满完成、加快“三市”建设的关键年。做好今年的工作，挑战与机遇并存，困难与希望同在。从宏观经济形势看：世界经济虽然保持缓慢复苏态势，但新的增长动力源尚不明朗；我国经济进入了增长速度换挡期、结构调整阵痛期、前期刺激政策消化期叠加阶段，长期向好的基本面虽然没有改变，但不稳定、不确定、不可持续的因素依然较多。这些因素都会对我市经济的发展产生一定影响。从我市发展的阶段性特征看：汉中正处于发展的关键时期。这个时期是保持一定速度、持续扩张总量、努力爬坡上坎的追赶跨越期；是突出循环发展、促进转型升级、不断提质增效的集中攻坚期；是突破交通瓶颈、彰显区位优势、全面开放开发的潜能释放期；是统筹各种资源、推进“四化”同步、增进群众福祉的民生普惠期。这种阶段性特征和我市经济后发型、自主内生性强的特点，将使汉中的发展呈现逆势上扬、稳中有进的良好态势。从有利因素看：中央全面深化改革、加快推进新型城镇化等重大战略部署，为我们破解发展难题、加速释放潜能，增添了强大动力；国家实施积极的财政政策和稳健的货币政策，为我们实现稳增长、调结构、促改革目标提供了有力保障；西部大开发、秦巴山片区扶贫攻坚的深入推进和丝绸之路经济带的启动建设，为我们全方位加快发展提供了广阔空间；国家生态补偿制度的进一步完善，特别是南水北调对口协作机制的逐步实施，为我们加快生态建设、争取多方支持搭建了平台；汉中机场、宝汉高速、西成客专、阳安二线等重大交通设施的建成，将大大提升汉中的区位优势，也使我们的资源优势、产业优势、生态优势得以更好地彰显出发展的前景和实力。总体上看，今后几年我市将同全省一样保持上升趋势，处于中高速发展挡位上。我们必须把握大势，拼抢机遇，奋发有为。

按照省委、省政府关于汉中“循环发展、生态宜居”的战略定位，围绕实现汉中“三市”建设目标任务，今后一个时期，我们要着眼全局、统筹兼顾，重点抓好关乎全市未来发展的“十大工程”和“五大园区（村）”建设。“十大工程”是：千亿投资工程、工业翻番工程、农业倍增工程、全域旅游工程、城乡统筹工程、文化振兴工程、生态环境工程、扶贫攻坚工程、民生改善工程、治理创新工程；“五大园区（村）”是：工业园区、农业园区、精品景区、新型社区、美丽乡村。基于上述战略考虑，2014年，全市经济社会发展的总体要求是：全面贯彻落实党的十八大和十八届二中、三中全会，中央经济工作会以及省委十二届四次全会、市委四届五次全会精神，围绕科学发展、建设“三市”主题，紧扣转方式、调结构主线，把握稳中求进、改革创新、提质增效的核心要求，以做强循环经济促进转型升级，以推进新型城镇化做优发展空间，以生态环境建设打造美丽汉中，以全面深化改革增强发展活力，以加快改善民生增进群众福祉，切实提高发展质量和效益，促进经济持续发展、社会和谐稳定。

按照这一要求，今年经济工作的主要预期目标是：生产总值增长12%左右，全社会固定资产投资增长25%以上，规模以上工业增加值增长19%，地方财政收入增长13%，社会消费品零售总额增长15%，城镇居民人均可支配收入和农民人均纯收入分别增长12%、13%，居民消费价格指数、城镇登记失业率、人口自然增长率分别控制在4%、4.3%、5‰以内，万元生产总值能耗下降3.3%。

实现上述目标，必须把握好以下原则：一是坚持加快发展不动摇，争先进位求突破。牢牢抓住发展第一要务，坚持稳中求进、好中求快、快中提质，自我加压，主动作为。二是坚持改革开放不停步，更新理念抢先机。以解放思想为先导，用改革破解难题，靠创新激发活力，全面落实深化改革的各项政策措施，更多更好地释放改革红利和发展潜能。三是坚持转变方式不松劲，加快转型促升级。围绕提高经济增长质量和效益，大力改善需求结构，优化产业结构，强力推进循环发展。四是坚持生态立市不懈怠，彰显优势拓空间。始终把汉中的绿水青山作为发展的最大优势，着力构建集约高效的生产空间、宜居适度的生活空间、山清水秀的生态空间。五是坚持改善民生不止步，共享成果增福祉。全面落实各项惠民政策，大力推进基本公共服务均等化，把汉中建设成为宜居宜业、民富民安、公平公正、和谐和美的幸福家园，让人民群众生活得更有尊严和自信。

三、2014年工作重点

做好今年工作，任务十分艰巨。我们将按照市委四届五次全会的安排部署，紧扣重点，整体推进。

（一）突出重点项目建设，持续扩大投资规模

大力实施“千亿投资工程”。充分发挥重点项目的支撑引领作用，全年实施重点项目和重大前期项目212个、年度投资314亿元，确保全社会固定资产投资完成849亿元，力争“十二五”末全社会固定资产投资超过1000亿元。

抓项目推进。继续坚持项目建设三项制度，建成宝汉高速汉中以南段路基工程、佛坪陈家坝至三河口道路改建等项目，开工建设宝汉高速汉中以北段及留坝连接线等工程，加快西成客专、云河水库和大唐略电扩建、国电电网改造等项目进度，继续抓好阳安二线、宝南铁路等项目前期工作。

抓谋划争取。把握中、省政策导向，加大项目前期投入，精心策划筛选一批科技含量高、经济效益好、资源消耗低、环境污染小，尤其是与南水北调水源保护、城市基础设施相关的大项目、好项目，千方百计挤进中省盘子，为持续发展提供有力支撑。

抓招商引资。强化项目包装和活动策划，积极承接东部产业转移，在充分利用中省招商活动平台的同时，精心组织好赴长三角、珠三角、环渤海等地的招商引资活动。密切关注国内500强企业等大集团的投资趋向，加大叩门招商、以商招商力度，寻求合作意向，积极拓展与中交、中航、陕煤、陕旅、碧水源、通用航空等大企业的合作，不断提高引资引智成效，全年引资到位完成356亿元。

抓服务保障。扎实开展土地清理，整合利用闲置土地，保证建设用地需要。积极搭建银企合作平台，引导金融机构扩大信贷投放，发挥投资主渠道作用；进一步激活民间资本，多方筹措项目建设资金。建立联审联批工作机制，加大工程建设领域环境整治力度，确保项目建设顺利实施。

（二）做大做强产业集群，努力推进新型工业化

大力实施“工业翻番工程”。抓技改、强支柱，解难题、保增长，继续做大做强装备制造、新型材料、农副产品加工三大产业集群和其他骨干产业，确保全年规模以上工业总产值突破1000亿元。

强化协调服务。坚持工业经济目标考核机制，抓好工业促销稳产保市场各项政策落实；强化监测分析，及时解决生产经营中的困难和问题，促进骨干企业满负荷生产，确保规模以上工业增加值达到320亿元。

强推工业项目。实施工业重点项目80个、完成投资55亿元。确保汉中卷烟厂、秦洋长生酒业、西乡秦自然生物提取、略阳3万吨电解锰等改扩建项目建成投运，汉机公司高新技术产业基地、科瑞思高品位石英、亚特电器高压配套、燕航重卡发动机配套、陕飞中型运输机扩能改造等项目加快推进，堂宏集团客车生产线、洋县3万吨金属钙生产线等项目开工建设。

强力打造园区。以汉中经济技术开发区、汉中航空智慧新城、洋县有色冶金产业园和县区工业园区为重点，加快完善基础设施，增强项目承载能力，吸引企业集聚发展，培育一批规划科学、特色鲜明、链条完整、节能环保、竞争力强的产业集群。全年力争新创建省级工业园区4个，新入园企业40户以上。

强抓非公经济。坚持权利、机会、规则“三平等”，鼓励非公资本控股或参股混合所有制企业，激发非公经济活力和创造力。加快推进中小企业服务体系建设，新发展小微企业1500个，培育规模以上企业超过30户。促进品牌、商标带动，力争新增1个中国驰名商标、30个陕西著名商标、6个省级名牌产品、2个省级以上知名品牌园区。

（三）积极发展现代农业，不断提升综合生产能力

大力实施“农业倍增工程”。以中央农村工作会议精神为引领，加快农业现代化，稳步提高传统农业规模效益，突出抓好林下经济、水产养殖业，力争用五年、七年时间，分别使农民人均纯收入、农业总产值在2012年基础上实现倍增。今年实现农业总产值344亿元、农民人均纯收入达到8000元。

夯实农业基础。实行最严格的土地保护制度，划定耕地保护红线，稳定粮油种植面积。加强农田水利基本建设，提高农业抗御水旱灾害能力。加大农业先进适用技术推广应用和农民技术培训力度，加快发展现代种业。不断提升农业设施化、机械化水平，促进农业增产增效。

做强产业基地。围绕“三个六”、“四个优”、“七个特”主导产业，加快发展以粮油、茶叶、畜牧、蔬菜、水果为重点的有机农产品基地，以名优特品种养殖和冷流水养殖为重点的水产品基地，以用材林、干果经济林、木本油料及中药材、苗木花卉等为重点的林产品基地，推进农业标准化、规模化发展。全年粮、油总产分别稳定在100万吨、17万吨，生猪出栏430万头，茶叶、水果产量分别达3.2万吨、42万吨，蔬菜产量稳定在200万吨左右，水产品产量3.45万吨。

建好现代园区。强化龙头企业带动，加速土地、设施、科技等要素聚集，着力培育一批优质高效、生态安全的现代农业园区。全年新创建市级以上农业园区24个、市级渔业示范园10个，抓好11个市级林业产业示范园建设。

培育经营主体。深化农村改革，不断探索农村土地集体所有制的有效实现形式，落实集体所有权、稳定农户承包权、放活土地经营权，加快构建以农户家庭经营为基础、合作与联合为纽带、社会化服务为支撑的现代农业经营体系，引导农村实用人才、致富带头人、返乡农民工大胆创业、规模经营，大力培育职业农民、专业大户、家庭农场、龙头企业和专业合作社。全年新发展市级以上龙头企业20户、农民专业合作社200个。

保障农产品安全。加快推进县镇食品、农产品质量安全检测体系和监管能力建设，落实属地管理和生产经营主体责任，完善农产品质量和食品安全工作考核评价制度，用最严格的标准、最严密的监管、最严厉的处罚、最严肃的问责，建立健全覆盖从田间到餐桌全过程的监管制度和责任追究制度，确保广大群众“舌尖上的安全”。

（四）壮大旅游商贸经济，加速发展现代服务业

大力实施“全域旅游工程”。依托以“两汉三国”为主要内容的历史文化和秦巴山水优质生态资源，精心打造精品旅游产业，拉动服务业加快发展。

加快旅游产业发展。精心编制《汉中全域旅游发展规划》，深入推进“一江两岸”景区、龙岗文化生态旅游园、西汉三遗址等项目建设；支持洋县创建省级旅游示范县，加快华阳、紫柏山景区创5A和黎坪、青木川、五龙洞、午子山、武侯祠、朱鹮梨园景区创4A步伐；推进旅游特色名镇、旅游示范村和星级农家乐建设，提升乡村旅游发展水平；加强旅游宣传推介，办好系列营销活动，全年接待游客突破2500万人次、旅游总收入130亿元。

做强商贸物流产业。加快褒河物流园、亿丰商贸城等项目建设，健全社区便民服务网络、再生资源回收体系；精心实施“新网工程”、“镇超工程”、“农超对接工程”。全年培育限额以上企业30户以上；大力培育休闲、健康、养老、家政等新业态和汽车、信息、电子商务等消费新热点，全年社会消费品零售总额完成285.32亿元。

全面扩大对外开放。抢抓国家建设丝绸之路经济带的历史机遇，及早谋划、主动融入，加快向西开放步伐；加快汉中海关建设，健全口岸服务功能；实施“外贸龙头企业培育”和“外贸孵化器”工程，大力培育外贸进出口企业。全年进出口总额增长10%以上。

（五）坚持城乡一体化发展，建设生态宜居城市

大力实施“城乡统筹工程”。坚持“以人为本、四化同步、科学布局、绿色发展、文化传承”五大原则，紧扣“建城、兴业、社保、管理”四项任务，着力打造以中心城区为核心，以沿江七个县城为节点，以沿江重点镇为单元，以汉江为纽带的串珠式滨江城市群和城镇带，加快建设生态宜居城市。

精美规划彰显特色。统筹考虑整体建筑风格、水景绿化体系、历史遗迹保护、民俗文化传承，全面推进新一轮城镇总体规划、专项规划和重点区域控制性详规修编，着力打造一批体现山水人和谐相处、彰显民俗风情、传承地域文化的特色城镇。特别是中心城区要围绕历史名城、生态宜居主题，实施“西进做大、东扩做强、南移做精、北优做特、老城提升”战略，体现“五大城市形象设计”，即：以秦巴汉江秀美山水为表现元素，体现生态城市的山水之美；以两汉三国文化元素的挖掘弘扬，体现历史文化名城的人文魅力；以巴蜀文化特别是川陕民居民俗为元素，体现古朴灵秀城市的地域特色；以时尚社区的高品质开发，体现现代城市的时代气息；以组团式的空间体系，体现田园城市的发展格局。

精品打造整体推进。中心城区建设上，加快滨江新区、兴元新区、江南片区、西汉三遗址和东关、南关正街的建设开发；建好梁州路、天汉文化公园、丝绸大道、翠屏路、傥骆路等项目；开工金牛路、沙沿路、东五路等项目，不断完善功能、提升品位，充分发挥中心城区的示范带动辐射作用。县城建设上，以完善功能、改善环境、凸显特色为重点，强化产业支撑，完善公共服务，提高承载和带动能力。大镇名镇建设上，发挥好2个省级重点示范镇、5个省级文化旅游名镇和15个市级重点示范镇带动作用，整体提升集镇建设水平。村庄建设上，大力开展“美丽乡村”和“新型农村社区”创建活动，打造环境优美、生活甜美、社会和美的新农村。

精细管理提升质量。以人的城镇化为核心，把有序推进农村转移人口市民化作为新型城镇化的首要任务，把农村居民就地市民化作为城镇化的主要途径，大力推进以土地、户籍、教育、医疗、社保制度为主要内容的配套改革，全年实现农村居民进城落户10万人。提升城镇绿化、亮化、净化、美化水平，实现省级园林县城全覆盖和佛坪、镇巴县创国家园林县城目标，中心城区创建国家卫生城市通过综合评审。

（六）落实功能区域规划，强力发展县域经济

大力实施“扶贫攻坚工程”。抢抓秦巴山片区扶贫攻坚机遇，满足生态功能区规划的刚性要求，支持县区进一步发挥区位优势、资源优势和产业优势，走特色发展、错位发展、协调发展的路子。

做好扶贫开发。坚持科学规划、因地制宜、抓住重点，扎实推进专项扶贫、行业扶贫、社会扶贫，提高扶贫工作的精准性、有效性、持续性。以146个村的整村推进、5.8万亩高标准农田建设为抓手，改善基础设施条件，提高综合生产能力，促进农民增收脱贫。

做强中部龙头。汉台区要壮大园区工业，繁荣商贸物流，提升特色农业，建设陕西强区；南郑县要扩张工业规模、打造精品旅游、补齐三产短板，建设省级强县；开发区要抢抓扩区调整机遇，全力招大引强，发挥好国家级经济技术开发区的辐射带动作用。

做大东西两翼。城固县要坚持县城和航空新城“双核互动”，加快释放潜能，壮大县域实力；洋县要抓好生态农业、循环工业、精品旅游，争创陕南循环发展、生态宜居示范县；勉县、略阳县要更加注重资源深度开发、环境保护、城乡统筹和城市管理，不断推进县域经济转型升级。

做优循环示范。西乡县要走资源高效开发、绿色低碳循环、经济特色发展路子，彰显富裕和谐优美茶乡魅力；宁强县要以循环经济、旅游商贸、城乡建设为重点，提升实力、实现跨越；镇巴县要坚持短抓绿色产业富民、长抓旅游产业蓄力、强抓油气开发突破，实现县域经济大发展。

做美特色亮点。留坝县要坚定一业突破理念，推进旅游产业提速发展，全域打造旅游名县；佛坪县要发挥生态资源优势，建设精美、宜居、富裕、和谐的山水家园，迎接高铁旅游经济的到来。

（七）加强生态环境建设，倾力打造美丽汉中

大力实施“生态环境工程”。扎实推进汉江流域污染防治三年行动计划和省级生态文明建设示范市、环境保护模范城市创建活动，为美丽汉中建设奠定坚实基础。

突出循环发展。坚持减量化、再利用、资源化，大力发展资源节约型、清洁生产型、生态环保型产业；积极引进、开发新能源和可再生能源项目，加快培育一批循环经济示范企业、示范基地和示范园区。以工业、建筑和交通运输、公共机构领域节能为重点，加强电力、钢铁、有色冶金、建材等高耗能行业和重点耗能单位的节能管理；严格落实相关政策，坚决限批高耗能项目，加快淘汰落后产能。

加强环境保护。围绕“绿水、蓝天、青山、宁静”目标，打好环境治理“四个战役”，集中抓好汉江流域综合治理，大气污染、重金属污染防治和农村生态环境连片整治，下功夫治理露天焚烧秸秆、城区燃煤、建筑工地和街道扬尘、城市噪声、超标排污、废旧回收企业二次污染等突出问题。划定重点区域产业负面清单，坚决防止新的污染源产生。中心城区污水、垃圾集中处理率分别达90%、97%，县城污水、垃圾集中处理率分别达77%、86%以上，汉江出境水质始终保持在二类以上，中心城区空气质量二级以上优良天数保持340天以上。

推进生态建设。围绕城镇森林化、村庄社区园林化、三边景观化、门户亮丽化，持续抓好城镇街道、单位小区、交通干道、江河库渠沿岸绿化，精心实施天然林保护、退耕还林、长江防护林、森林抚育等重点工程，全年完成造林18万亩，实施街道、交通干线及河堤绿化365公里，治理水土流失850平方公里。

（八）提升公共服务水平，全面发展各项社会事业

大力实施“文化振兴工程”。围绕建设文化名市目标，积极倡导和践行社会主义核心价值观，弘扬主旋律，聚集正能量，努力推进文化振兴。按照统一谋划、分步实施的要求，逐年启动实施市博物馆、图书馆、科技馆、群众艺术馆、青少年宫、老年活动中心、游客服务中心、体育运动中心、会展中心和汉中大剧院10大公共文化设施建设；深入挖掘地方民俗、特色文化资源，搞好镇巴民歌、汉调桄桄等文化遗产的保护传承，策划创作一批有影响力、吸引力的文艺作品、舞台精品；抓好基层文体设施建设和惠民活动开展，活跃群众文化生活；引进战略投资者重组和提升我市文化企业，推动文化与旅游融合发展。全年新培育文化企业100户，文化产业增加值增长30%。

大力实施“民生改善工程”。把“守住底线、突出重点、完善制度、引导舆论”作为改善民生的基本遵循，切实抓好民生政策衔接和落实，让群众更加公平地享受发展成果。认真落实各项就业优惠政策，鼓励全民创业，以创业带就业，重点做好高校毕业生就业和淘汰落后产能中出现的下岗人员再就业工作。依托市职业技术学院等院校，开展对贫困家庭和移民搬迁户适龄子女的正规化职业教育，使其通过教育实现稳定就业。全年新增就业2.9万人，转移农村富余劳动力75万人。认真做好农民工、非公经济组织从业人员等群体参加职工社会保险工作，全面落实城乡一体化的居民社会养老保险制度。认真落实孤残儿童、高龄老人补贴政策，加大临时救助和医疗救助力度，加强社区管理和服务，大力扶持各类养老机构，鼓励兴办社会养老休闲产业。继续抓好“三项重点工作”，全年新修、加固汉江干支流堤防40公里，新开工保障性安居工程3.52万套、建成2.12万套，移民搬迁2.2万户8.36万人。深化教育领域综合改革，促进城乡义务教育均衡发展；扎实推进“双高双普”，西乡、留坝建成“双高双普”县；完成中心城区学校建设攻坚工程，新建扩建学校8所，支持陕西理工学院创建综合性大学；创新人才培养、引进机制，培养高素质劳动者和技能型人才。深化县级公立医院改革，推进医疗卫生服务县镇村一体化管理，加强基层医疗卫生服务能力建设，建成市级公共卫生应急指挥中心和全科医生培训基地，继续抓好城乡居民大病保险试点工作。积极适应计生政策调整，落实好奖励扶助政策，持续稳定低生育水平。推进政府购买服务，发挥社会组织在发展民生中的积极作用。精心组织城乡居民收入调查一体化改革，认真做好第三次经济普查和二轮修志工作。

大力实施“治理创新工程”。顺应国家治理体系和治理能力现代化发展大势，努力推进社会治理创新。以保障和改善农村民生为优先方向，以解决农村“三留守”、空心村问题和化解社会矛盾、加强基层组织等为重点，强化农村社会管理。以实施网格化管理为抓手，不断提升城市社区管理水平。加大重点行业、重点场所、重点部位和重要时段安全生产隐患排查整治力度，坚决杜绝重特大事故、遏制较大事故、减少一般事故。加强普法宣传和法制教育，提高社区矫正管控、帮教、转化水平，建设法治汉中。围绕“抓三强、攻三案、提三率”，全力推进领导干部接访、约访、下访，努力提高初信初访化解率，及时就地解决群众的合理诉求。高度重视并及时解决民族宗教领域的热点、难点问题。健全完善社会治安防控体系，深入推进社会治安综合治理，加大严打整治和“黄赌毒”治理力度，增强人民群众安全感，建设平安汉中。

为了让改革发展成果更好地普惠于民，市政府承诺今年为城乡群众办好“十件实事”：一是建成农村公路560公里；二是建成标准化学校100所；三是将新农合和城镇居民医保政府补助标准提高到人均350元；四是建成农村互助幸福院100个；五是建成城乡体育惠民设施328处；六是建成基层文化活动场所380个；七是解决25万农村群众饮水安全问题；八是实现脱贫13万人；九是在汉江沿岸7个重点镇开工建设垃圾、污水处理设施；十是在中心城区办好便民利民“10件好事”。

四、切实加强政府自身建设

面对新形势、新任务、新要求，我们必须以贯彻党的十八届三中全会精神为统揽，以开展党的群众路线教育实践活动为动力，切实加强政府自身建设，不断提高执行力和公信力，努力建设人民满意的政府。

（一）大胆改革，建设创新型政府。以解放思想为先导，坚持用宽广的视野、开放的意识、创新的举措、市场的手段，积极稳妥地推进行政管理体制改革、农村改革、财税金融改革等一系列重大改革，攻克体制机制障碍，破解发展难题，使改革创新成为加快汉中发展、推进“三市”建设的不竭动力。

（二）勇于担当，建设高效型政府。牢固树立勇于担当、奋发有为、争创一流的进取意识，在重点工作、难点问题、关键环节上勇挑重担、敢抓敢管。大力发扬踏石留印、抓铁有痕的拼搏劲头，看准的事情盯紧不放，定下的工作抓紧落实，善谋善为，善做善成。大力推进简政放权，最大限度减少行政审批，同步规范前置审批，促进审批再提速、流程再简约、服务再优化。

（三）规范有序，建设法治型政府。自觉接受市人大法律监督、市政协民主监督和新闻媒体、人民群众的社会监督，精心办理人大代表建议和政协委员提案。完善政府重大事项专家咨询、集体决策、社会公示制度，努力使政府决策更加科学民主。深入推进政务公开、信息公开，重点搞好部门预算公开和“三公”消费公开，防止权力滥用和暗箱操作。强化行政执法监督，促进公正文明执法，维护社会公平正义。

（四）执政为民，建设服务型政府。扎实开展党的群众路线教育实践活动，进一步密切同人民群众的联系，始终秉持为民情怀，主动深入基层一线，察实情、出实招、办实事、求实效。充分利用公民代表进机关、市长热线、市长信箱、网络问政、来信来访等渠道，广泛听取人民群众意见建议，及时解决群众反映的困难问题。

（五）从严治政，建设廉洁型政府。严格贯彻落实中央八项规定，大力反对“四风”，坚决停止新建和改扩建政府性楼堂馆所，严控机构编制和人员，确保“三公”经费和会议费只减不增。突出搞好工程建设项目招投标、政府采购、国有土地使用权出让等领域的专项治理，加强反腐倡廉基础制度和公共资源交易平台建设，严肃查处各种违法乱纪行为和腐败案件。切实加强行政监察、审计监督和预算管理，努力实现对公共资金、国有资产、公共资源审计监督全覆盖。

各位代表，汉中已进入大建设、大发展、大跨越的新阶段。让我们在市委的坚强领导下，团结和依靠全市人民，进一步解放思想，求实创新，扎实工作，以时不我待的紧迫感和舍我其谁的奋发精神，全力推动经济社会又好又快发展，为早日实现“三市”建设目标而努力奋斗！
